# Replacing Text with Pictures for Multi-Lingual Health Education: Meeting the Needs of a Community with Low Literacy in Tanzania

**DOI:** 10.3390/ijerph22040516

**Published:** 2025-03-28

**Authors:** Jeremy C. Barrett, Jaya A. R. Dantas

**Affiliations:** Curtin School of Population Health, Faculty of Health Sciences, Curtin University, Bentley, Perth, WA 6102, Australia; jerrybarrett2@icloud.com

**Keywords:** health literacy, literacy, pictures, education, healthcare, cartoons, community, hygiene, prevention

## Abstract

Rural and remote communities often face significant challenges maintaining their health and well-being. In developing countries, these challenges are further compounded by (1) limited access to clean water, (2) ineffective sanitation, (3) low education and literacy levels and (4) low awareness of the role of personal hygiene practices in reducing communicable disease transmission. Existing health education materials distributed within such communities are often presented in a traditional written format, making them inaccessible to people with low literacy levels. Therefore, recent evidence supports an alternative health communication approach through the use of picture-based materials. This study has assessed the effectiveness of a cartoon-based pictorial educational tool (CBPET) in communicating key messages about hygiene and bacteria contained within the environment and bodily fluids. The CBPET that was developed was tested in a Tanzanian village, representative of a community with low literacy and a resource-poor setting. The CBPET was found to effectively deliver key health promotion messages to the local community. Developing healthcare education in a universal language format based on pictures or cartoons could be the way forward.

## 1. Introduction

The ability of healthcare messages to effectively educate vulnerable groups is a vital component towards improving public health. Despite the literacy rates in children showing a welcomed increase since this initial research [1], the ability to communicate important health-promotion material is still low among older generations in many parts of the world who have limited literacy [2,3]. This is due to much of the current healthcare education, information and instructions utilizing a written format [2,3]. This research has indicated that a well-designed cartoon-based pictorial educational tool (CBPET) may be an effective universal method to deliver healthcare messages equitably to all members of the community.

### 1.1. Pictorial Communication and Literacy Skills

Low health literacy acts as a barrier to individuals’ ability to participate effectively as productive citizens, as it limits their ability to understand rules, regulations and public health notices [4,5]. Health literacy can be defined as “the degree to which individuals have the capacity to obtain, process, and understand basic health information and services needed to make appropriate health decisions” [6].

Health literacy is closely linked to health promotion, as it emphasizes the need for individuals to have sufficient literacy skills not only to read information, but also to critically analyze and understand it. It also involves overcoming structural barriers that stem from the social, economic and physical conditions in which people live [5,7]. The focus on overcoming structural barriers is also centered around empowerment, where individuals are enabled to access, understand, critically evaluate and effectively apply the health information in their daily lives [7]. The community’s level of textual literacy often dictates the medium for communicating healthcare messages. Therefore, choosing appropriate communication modes for a community is vital, as the use of inappropriate modes can result in disempowerment and health-related information being unsuccessfully delivered [7,8]. More innovative approaches to health communication are warranted [8,9].

Prior to the introduction of the written word, many Indigenous communities throughout history have shared life experiences and skills through pictographs and stories [10,11]. These alternative modes of communication have been evidenced as effective in the transmission of information [12,13,14]. In fact, the use of pictures or visual aids in promoting health messages and health literacy is not unusual and has been used since the 1860s [15]. Research documents the effectiveness of using cartoons in health promotion, such as in sexual health [16], disease management [17] and health-related behavior changes (e.g., eating habits) [18] or COVID-19 preventive responses [19].

One of the earlier studies that examined the effectiveness of visual health communication was that of Delp and Jones, who investigated the use of cartoons in delivering wound care information to patients receiving treatment within a United States healthcare facility [20]. The sample group was not chosen based on literacy levels; however, subsequent analysis revealed that out of the 234 participants enrolled in the study, 57 individuals, all of whom had less than a high school education, successfully followed the cartoon-based instructions and reported high satisfaction with the tool’s format [20]. In the same study, parents of child participants under 14 years of age were equally “very satisfied” with the children’s ability to understand and comply with the cartoon-based information compared to the written format used previously [20]. Therefore, the study highlighted how using pictorials or images is an effective way to deliver information to all age levels within the community, regardless of textual literacy competence [20]. Specifically, using pictures in health information has been shown to considerably improve knowledge, understanding and recall amongst individuals with lower health literacy [13]. These findings have been further supported by research and a number of scoping reviews and meta-analyses in recent years [12,13,14].

### 1.2. Public Health Education

Public health education is well established in many developed nations, with written manuals, reports and circulars as the main modes of information delivery. However, these communication modes are inequitable, as they are inaccessible to communities with low literacy levels [21]. According to UNESCO data from 2016, around 750 million adults were suffering from illiteracy, with women accounting for a large percentage of this number [22]. This highlights that a large proportion of the global population is unable to read and understand health education materials produced by most governments and health agencies, increasing the health disparity among communities [22].

Interestingly, low health literacy is not just associated with developing countries, as many Indigenous and Non-Indigenous communities within developed countries such as Australia and the United States are evidenced to have poor adult literacy skills [23,24]. One study in North America found that 36% of adults had inadequate health literacy skills when exploring health literacy and obesity among Native Hawaiians and Pacific Islanders [25]. Additionally, data from the Australian Bureau of Statistics found that only 39% of respondents found it easy to understand health information well enough to enable them to make informed decisions [26]. Despite advancements in medical research and healthcare systems, many communities worldwide lack the basic literacy skills required to access and utilize vital healthcare information and modern health services [22].

### 1.3. Basic Hygiene and Health

Basic hygiene, along with health literacy, are key foundations for achieving good public health outcomes and have been evidenced to reduce rates of infection [27,28]. Basic hygiene and infection control form the core of most health education systems, and understanding effective personal hygiene practices is a vital primary health action in the prevention of communicable diseases [29,30]. Contaminated hands play a key role in the transmission of fecal germs via the hand–mouth route [31,32]. Handwashing with soap (HWWS) can protect against the spread of infectious diseases, such as the Schistosoma infection, without any form of immunization [33,34]. There has been considerable research conducted to establish the link between a lack of effective hand hygiene and the spread of disease and poor health issues in developing communities [34,35,36]. Specifically, it has been found that HWWS is estimated to reduce morbidity due to acute respiratory infections by about 17% [7]. This figure suggests that many lives could be saved through appropriate health communication around personal hygiene practices. HWWS is one of the most cost-effective prevention strategies to prevent infectious disease transmission in developing communities [34,37].

Although HWWS is considered an effective prevention strategy, Curtis et al. found that only 17% of the study group from 11 developing countries, including Tanzania, washed their hands with soap after going to the toilet [38,39,40]. The study found that Tanzania had a high prevalence of gastrointestinal and respiratory infections, concluding that this was due to drinking water contamination via the fecal–oral route and inadequate hand hygiene [40]. Additionally, the death rate from disease and infection in many African countries is unacceptably high, with infants and women amongst the most susceptible [41,42,43]. Diarrheal and respiratory conditions are some of the leading causes of worldwide morbidity and mortality, particularly amongst children [44,45]. This is evidenced by data from 2021 that found that there were more than 400,000 deaths from diarrhea caused by 12 pathogens, with about 99% of these deaths occurring in low-income and middle-income countries [45]. The global issues of human immunodeficiency virus (HIV) and communicable infections (e.g., cholera, diarrhea, dysentery, influenza and sexually transmitted diseases) play a major role in this mortality rate [46,47]. These high death rates are highly preventable, with a lack of basic textual literacy being a significant influencing factor [27,48].

Hygiene is vital to good health, and identifying routes of transmission in the home and community settings is critical in breaking the chain of infection [31,35]. Therefore, recommendations for action are centered around preventative measures. One recommendation is that children should be taught about germs and HWWS from an early age to develop “social manners” or to enhance their disgust of “dirty” hands and thus improve the intrinsic behavioral practice of washing hands following key interventions [49].

### 1.4. Role of CBPET in Communities with Low Literacy

Though there is vast research on the effectiveness of CBPET in health literacy and health promotion, further research is needed on its effectiveness in low-literacy communities [14]. As communities with low literacy tend to be more culturally diverse, the CBPET developed for these communities needs to be culturally specific and contextually relevant. Research is also needed to evaluate the effectiveness of CBPET in communities who have low literacy levels and live in resource-poor settings [14]. In addition, social determinants of health, such as access to health resources and health information, literacy levels and income, all play a role in promoting healthy behaviors for disease prevention [7]. Hence, this research posits the need to explore the effectiveness of CBPET to promote health and improve health knowledge and awareness in this community.

## 2. Materials and Methods

### 2.1. Study Site

Tanzania is a country in East Africa which is classified as lower-middle-income. In recent years, the country has made significant social and economic progress; however, there is still progress to be made [50]. From 2014 to 2021, the net enrollment rate of children in schools increased by 8.4% in primary schools and 14.3% in secondary schools [50]. There has also been an increase in literacy rates, as in 2021 approximately 76% of the population was literate, a 6.2% increase from 2014 [50]. However, there are disparities between urban and rural areas of Tanzania, with the literacy rate being significantly lower in rural Tanzania, at 69.9% [50]. Clean water and sanitation are also major issues in the country, with approximately only 44% of households having access to basic sanitation facilities and access to clean water ranging from 50% to 65% of households, depending on the season [50]. However, again, there are clear inequities between rural and urban areas. Over a 30-year period, from 1990 to 2021, Tanzania’s Human Development Index (which encompasses health, knowledge and the standard of living in a country) increased by 48%, where it now stands at 0.549 [51]. Tanzania is now ranked 160 out of 191 countries, and this score is above the average for countries in sub-Saharan Africa [51]. Improvements in income, health, living standards and education have been evidenced as key drivers of this increased score.

The specific study sites chosen for the research were the villages of Mlegele and Masanganya, in the District of Kisawarei, Tanzania. When the research was conducted, these villages were chosen, as they met the criteria of being resource-poor and having low levels of literacy among the general population. These small villages are typical of rural communities in eastern Tanzania, with low access to clean water, adequate sanitation, electricity and quality healthcare [50].

### 2.2. Study Design

The aim of this study was to record the change in awareness towards the importance of HWWS through the use of CBPET rather than assessing long-term behavioral changes. The research was conducted in three phases. It used a mixed-method approach that included semi-structured interviews, pre- and post-facilitation questionnaires and the CBPET.

Phase 1 consisted of the final development of materials, including the CBPET, preand-post questionnaires and semi-structured interview schedule, as well as obtaining ethics approval at the university. Additionally, the first author facilitated the introduction of donated ambulances and provided some training for use in the villages as part of a larger funded project, along with the delivery of a training package to village healthcare workers. This aided in the researcher’s introduction and in gaining the trust and confidence of the study cohort and community elders.

Phase 2 included recruitment of trial volunteers, implementation and delivery of the CBPET and data collection. The pre- and post-delivery questionnaires were completed by participants 24 h apart to ensure that the CBPET content was fresh in the participants’ memories. The results and comparison of the pre- and post-intervention questionnaires provided information on the effectiveness of the CBPET within the study group.

Phase 3 was data analysis and interpretation. Data collected were initially transcribed in Swahili and then translated into English for qualitative interpretation and analysis. All translation work was conducted by the Global Health Alliance of Western Australia (GHAWA) project manager and translator.

### 2.3. Questionnaire Design

A control group was not practical due to limited resources and funding available for the field trial, low numbers of eligible participants and time constraints for venue use. A one-group “pre-test, post-test” design was utilized to provide a snapshot of the data and to enable comparisons of pre-knowledge and post-knowledge.

The two-stage questionnaire was largely quantitative. However, open-ended and semi-structured interview questions were incorporated to provide a better understanding of the issues faced by a community with low literacy. Qualitative data assessed the participants’ knowledge of common pathogens, infection routes and transmission, frequency and motivation of HWWS and perception of the CBPET. This information can hopefully assist in designing global educational content for comparable communities that may benefit from a similar model.

Both questionnaires collected participants’ demographic information, knowledge about hand hygiene and feasibility of the CBPET tool (example question: what is a germ?). Participants were to respond to one of the three options: yes, unsure, or no. Participants were prompted to elaborate via written words or verbally. For example, when participants responded yes to the example question, they were further prompted to “describe a germ in your own words”.

The pre-intervention questionnaire aimed to establish a baseline of knowledge, and following the CBPET intervention, the same baseline questions were repeated in the post-intervention questionnaire but with some additional questions. The questions were further explained by a translator, with the opportunity for the participants to ask questions should they not understand what was being asked of them.

### 2.4. CBPET Design

The CBPET was initially proposed to be short, at four pages or less, simple, to engage the reader, and cost-effective. The CBPET was designed to tell the pictorial story of the journey of a germ from host to recipient and to reinforce how the chain of infection is broken by HWWS. The tool aimed to communicate this message to a community with low literacy and poor resources as a first step in preventing the spread of disease and improving quality of life for a community unable to effectively read and understand traditional public health-promotion messages.

**Figure 1:**
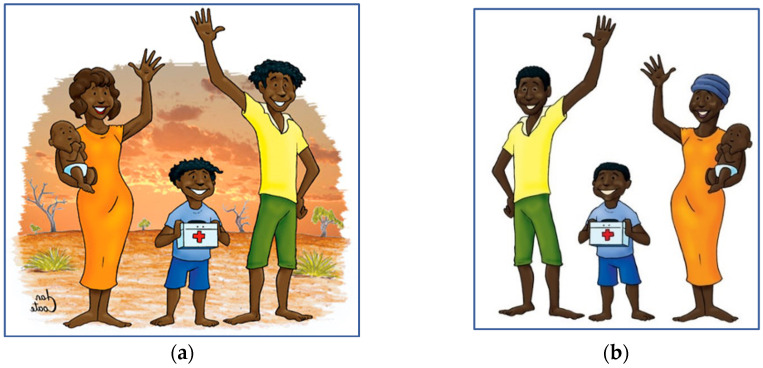
(**a**) Original artwork of the family group for the Australian Aboriginal version; (**b**) reconfigured artwork of the family group for the African version.

The first characters designed for the CBPET were a family consisting of a husband, wife, small child and infant (Figure 1). These characters indicated that any member of the family could assist in times of crisis and also attempt to reinforce community values. The family was initially designed to resemble Aboriginal Australians due to the intended target of the tool. In order to avoid narrowing the scope of the target audience to a particular tribal trait, the skin color, style of hair and type of clothing were symbolized to offer as little social impact as possible. As the scope of the research matured and ethical considerations were discussed, the intended study group shifted from a community with low textual literacy and poor resources within Australia to a small, remote community in Tanzania, East Africa. A majority of adults in the community had poor literacy skills, consistent with official Tanzanian statistical surveys on adult literacy [52]. The village elder welcomed the idea of increased education about “bugs” and “germs” for the community, and therefore, the ethnicity of the study population was altered to sub-Saharan Africa. Subsequently, the design of the human characters was changed, including altering the skin color, hair and clothing, to a generic sub-Saharan African appearance.

The next stage was to develop cartoon icons that would represent the various “bugs” in the CBPET that were “illness-causing germs”. The process included identifying germs that commonly spread illness and disease within developing communities, which resulted in five bugs being chosen.

The literature review indicated that some of the most common illnesses passed on via the fecal–oral route were respiratory or gastrointestinal [7,32,43,44,45,48]. This prompted the consideration of the respiratory-transmitted bacteria Streptococcus, water-transmitted virus hepatitis A, fecal-transmitted bacterium *Escherichia coli* (*E. coli*), fecal–oral- transmitted hybrid bacterium *E. coli* and the gram-negative, rod-shaped aerobic bacteria Pseudomonas. Character names were assigned to the bug icons to establish simple personas to be referred to during the story.

**Figure 2:**
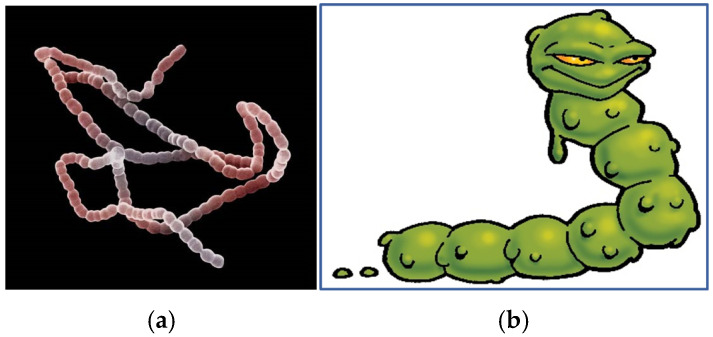
(**a**) Streptococcus bacteria (left) and (**b**) the phlegm bug.

The phlegm bug represented the gram-positive bacteria Streptococcus, which is a bacterium responsible for eye infections, meningitis and pneumonia (Figure 2). Its visual appearance was designed to look like a character that lived in the upper respiratory tract and was released from the body by the act of sneezing or coughing. This bug can then represent airborne pathogens commonly found in the upper respiratory tract, such as H1N1 virus, flu and the common cold.

**Figure 3:**
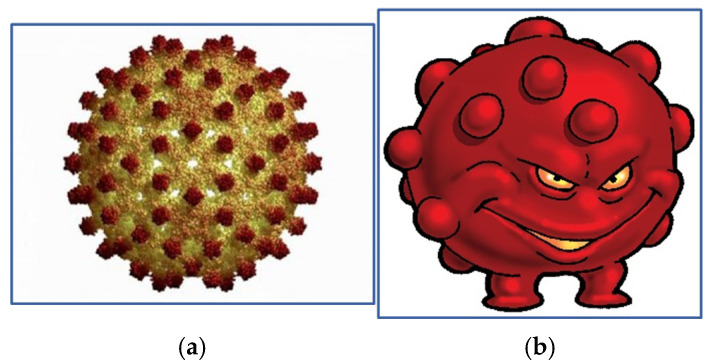
(**a**) Hepatitis B virus (left) and (**b**) the blood bug.

The blood bug represented the hepatitis B virus, a common cause of blood-borne infections in both developed and developing countries (Figure 3). The virus also bears physical similarities to HIV.

**Figure 4:**
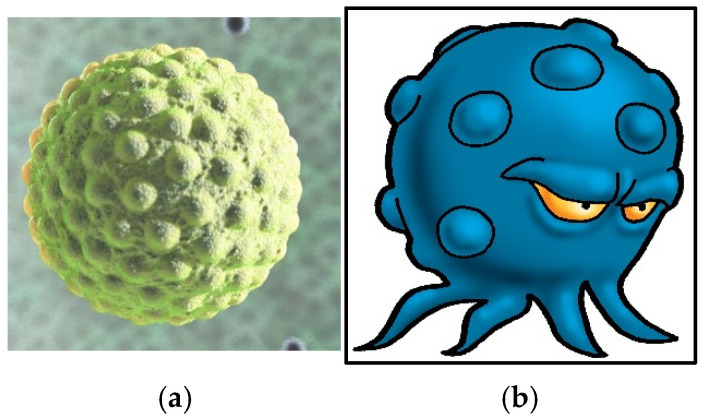
Hepatitis A virus (**a**) and the water bug (**b**).

The water bug represents the hepatitis A virus, which is commonly spread via the fecal–oral route and found in dirty or contaminated water (Figure 4). This virus is responsible for illnesses and death in developing countries. However, this icon was designed to symbolize dirty water and not solely hepatitis A.

**Figure 5:**
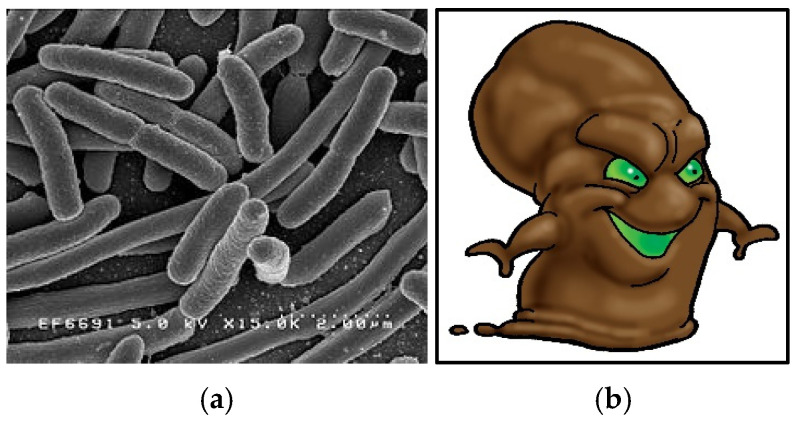
*Escherichia coli* (*E. coli*) bacteria (**a**) and the poo bug (**b**).

The poo bug represents *E. coli*, a bacterium commonly found in the lower intestinal tract of warm-blooded animals (Figure 5). This bacterium can cause food poisoning and serious illness and is also transmitted via the fecal–oral route.

**Figure 6:**
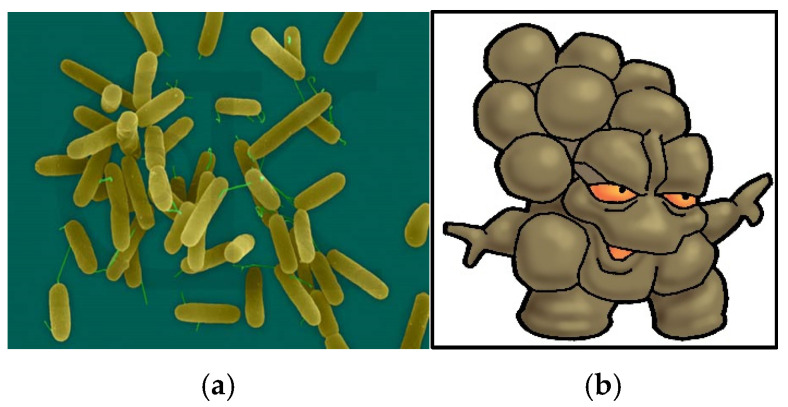
Mixture of Pseudomonas and *E. coli* bacteria (**a**) and the mud bug (**b**).

The last bug was a mud bug, a hybrid bug representing the pathogenic bacteria found in soil, mud and dirt (Figure 6). The bug was formulated from a mixture of *E. coli* and the gram-negative, rod-shaped aerobic bacteria Pseudomonas. The bug is a common precursor to wound infection and gastrointestinal illness, transmitted by both oral and topical routes.

### 2.5. Procedure

Participants were recruited by the village chief through purposive and snowball sampling. Inclusion criteria included participants being over 18 years of age. Initially, 22 participants were recruited. However, one participant dropped out, and one participant did not complete their pre-questionnaire; hence, their data were removed, leaving a total number of 21 participants (N = 21). The participants were provided with information about the project, and informed consent was obtained. Participants completed a pre-delivery questionnaire. The CBPET was then introduced to participants in a 20 min presentation by the first author and a translator. Participants were then asked to complete a post-delivery questionnaire 24 h after the pre-delivery questionnaire.

## 3. Results

### 3.1. Demographic Data

There were 21 participants (N = 21) who took part in the whole study, and the age range was from 25 to 75 years old, with a mean age of 45.8 years. Most participants were male (96%) and only four participants were female (4%). This disparity in gender distribution of participants was not intended in the recruitment process. The gender of the study group was not dictated by any part of the selection process. All members of the study group were married, which may be explained by the inclusion criteria that participants must be over the age of 18 and the cultural expectations of early marriage within the community. Eighteen (81.8%) participants identified as Muslim, two participants (9%) identified as Catholic, and one participant (4.5%) identified as Christian. One participant declined to answer. As a majority of the participants were Muslim, there was a gender imbalance, with women not attending the intervention.

A majority of participants (77.2%) had received primary school education only (up to year 7), three participants (13.6%) had received secondary education as well, and one participant had not received any formal education. Seventeen participants indicated that they were farmers, two participants indicated they were teachers, one participant described themselves as a “worker”, and another participant stated they were a driver. The questionnaire asked if participants had access to Western health services, and all participants (N = 21) stated that they had access to a Western medical clinic through an outreach program. The demographic data are summarized in Table 1.

### 3.2. Participants’ Hygiene Knowledge

#### 3.2.1. Participants’ Knowledge of Infection Cause, Infection Transmission and Hygiene Practice and Knowledge of Germs

In the pre-intervention questionnaire, all participants (N = 21) stated that they knew what a germ was. Six participants (27.2%) claimed a germ was a fly, three participants (13.6%) claimed it to be a mosquito, two participants (9.09%) stated a germ was a fly, a mosquito or a tsetse fly, and a further four participants (18.18%) indicated germs to be rodents. Another two participants (9.09%) stated that germs lived in dirty water, and one participant (4.5%) indicated a germ was a bed bug. The post-intervention results reveal an increased understanding amongst participants of what a germ is.

The number of participants identifying a fly as a germ halved from six (27%) to three (14%), but there was no change in the numbers of those who previously identified a fly, tsetse fly or mosquito as a germ (2; 9%) or who chose not to answer (4; 19%). Of interest are the four participants (19%) that stated a germ was a “fly bacteria”, “a fly spread by food” or “diseases (caused by) flies or mosquitos”. These responses indicate increased understanding and comprehension of bacteria and the spread of disease. One participant described a “worm” as a germ but may well have been trying to describe the “phlegm bug” (Figure 2) as a worm. If this was the case, it would indicate the “bug” image prompted an association with a germ and thus an understanding of the nature and possible identification of a “germ”, “bug” or bacterium.

#### 3.2.2. Participants’ Knowledge of Where Germs Live

In the pre-intervention questionnaire, only one participant (4.5%) claimed not to know where germs lived. The remaining participants gave the following answers: in a toilet (20%), in water (17%), in bushes/leaves (15%), in a poo (12%), on food (9%), in dirty water (3%), in dust (3%), in soil (3%), on a mosquito (3%). Five participants (15%) were also unable to explain where they were located and did not provide answers.

Following the delivery of the CBPET, those who could not explain where a germ lived and did not answer were reduced to two participants (6%). The location categories of where a germ lived also increased, with more study participants understanding that germs lived in water.

#### 3.2.3. Participants’ Knowledge of Germ Transmission

Data from the pre-intervention questionnaire indicate that the participants had a basic understanding of how germs were transferred between carrier and host; however, the scope of knowledge was limited. Participants were asked if they knew how germs travelled between infected people to others. Seventeen participants (77%) stated that they knew, three participants (14%) stated they were not sure, and two participants (9%) failed to answer. Following the delivery of the CBPET, the number of participants answering “yes”, they did know how germs travelled between people, increased to 19 (86%).

Participants who answered they were not sure were then requested to explain in their own words. In the pre-intervention questionnaire, fourteen participants (41%) stated that germs spread through the air, five participants (14%) through sneezing, four participants (12%) through touching hands, three participants (9%) through eating, two participants (6%) through insect bites and one participant (3%) through coughing. Five participants (15%) failed to answer, despite indicating they knew.

The concept of HWWS before and after sex sparked discussions amongst the study group, with a very comprehensive discussion ensuing around the “blood bug” character and how it invades the body. Analysis of the post-intervention data indicate that the concept of transmission of blood-borne disease was recognized by some of the group, with five participants (15%) stating that germs travel between people via sex, blood or wounds on the body.

#### 3.2.4. Participants’ Knowledge of Germ Infection

Participants were asked if they knew how germs enter the body. Those participants who answered “yes” were invited to explain in their own words. In the pre-intervention questionnaire, twenty-one participants (95%) stated they did know how germs entered the body, eleven participants (30%) answered “through the air”, ten participants (27%) indicated “through eating” and one declined to answer. The majority of participants understood the basic concept of germ transmission, which is via inhalation or oral route. However, the other answers suggest that there was some confusion with other disease-transmission routes. This may have been confused with the transmission of malaria and other mosquito-transmitted infections following large public health campaigns throughout the community.

After the delivery of the CBPET, the participants’ perception of how germs are transferred from reservoir to host improved, with all members answering “yes” and a broader range of answers provided. Participants better understood that germs may be spread from reservoir to host via “touching”, “coughing”, “sneezing”, “wounds” and drinking dirty water that had not been boiled.

#### 3.2.5. Participants’ Previous Education Experience in Handwashing

Participants were asked if they had ever been taught “how to wash their hands”. Prior to the delivery of the CBPET, 13 participants (59%) stated that they had received instructions on how to wash their hands, although there is no information on who delivered this. Eight participants (36%) stated that they had never been taught how to wash their hands, and one participant was not sure.

Following the presentation of the CBPET to the group, there was a large increase in certainty regarding the requirement for HWWS. Eighteen participants (82%) were now confident they had been taught how to wash their hands, despite the fact that this educational tool did not intentionally provide the methods of handwashing but merely highlighted the importance of it.

#### 3.2.6. Participants’ Understanding of the Importance of Handwashing with Soap

The pre-questionnaire asked participants if they thought handwashing was important. Nineteen participants (84%) indicated “yes”, it was important, and they were asked to explain why in their own words. Prior to the CBPET, there was a varied response, with six participants (27%) responding they washed their hands to avoid spreading disease, two participants (9%) to avoid disease and only one participant (5%) to kill germs. Other participants related handwashing with the removal of dirt or to make their hands look more presentable.

Following the intervention, all participants stated that handwashing was important, an improvement from the few who gave this answer in the pre-intervention questionnaire. Only two participants (9%) still stated that handwashing was to achieve cleanliness; the remaining participants explained handwashing was important to kill germs, avoid spreading disease and promote health.

#### 3.2.7. Participants’ Access to Soap

In the pre-intervention questionnaire, all participants were asked if they had access to soap in their daily routine. All participants (N = 21) responded “yes”, they did have access to soap. In the post-intervention questionnaire, there appeared to be some uncertainty, with one participant stating they did not have access to soap, and another participant was unsure. Following the delivery of the CBPET, a number of participants asked where they could access soap. The participants were then asked what they used to wash clothes at home, and all answered “with laundry soap”; it appeared participants were not aware that laundry soap could be used to wash hands. Several participants also wanted soap to be more available after knowing its importance in daily hygiene. This issue had been predicted by the translator prior to the delivery of the tool; therefore, soap had been purchased in advance. The soap was presented to the participants at the end of data collection.

#### 3.2.8. Participants’ Access to Water and Frequency of Handwashing

Access to a potable water source is vital to the ability to wash hands with soap and water and prevent infection via pathogenic infection. There were challenges in accessing potable water in the region.

In the pre-intervention questionnaire, participants were asked, “How many times a day do you wash your hands?” They were given four options: zero, one to three times, four to six times or more (seven+). Eight participants (36%) indicated they washed their hands one to three times a day, eleven participants (50%) indicated that they washed their hands four to six times a day, and only three participants (14%) indicated that they washed their hands seven+ times a day. Following the intervention, the number of participants stating their intention to wash their hands improved, with five participants (27%) intending to increase the frequency of handwashing to seven+ times a day. Most of those came from the group who reported handwashing one to three times a day in the pre-testing. The summary of participants’ hygiene knowledge is presented in Table 2.

### 3.3. Washing Hands

Participants were also questioned about the type of device used to hold the water when they washed their hands. The questionnaire offered the following choices: in a sink, in a bowl, in a stream, other (please state) or “I don’t wash my hands”. Fifteen participants (68%) stated that they used a plastic bowl, four participants (18%) named a “sink”, one participant (5%) indicated they used a stream, and two participants (9%) did not answer. One of the last questions in the pre-intervention questionnaire was, “Does soap and water kill germs?” Thirteen participants (59%) answered no, three participants (14%) were not sure, and only six participants (27%) answered yes to being aware of this fundamental hygiene fact.

The results of the same question in the post-intervention questionnaire suggest the CBPET effectively informed participants of the importance of HWWS in killing germs. The number of participants who stated soap and water do kill germs increased from six (27%) to nineteen (86%). This increase in knowledge is encouraging, but it does not explain whether the delivery of the message or the cartoon-based delivery format was solely responsible. Several other questions were asked to investigate whether the educational tool had significant impacts.

### 3.4. Impact of the Educational Tool

The morning following the delivery of the CBPET, additional questions were asked regarding participants’ knowledge and perception of hygiene and the impact of the CBPET. Behavioral change was an associated benefit of this educational tool; however, the main focus of this research was information delivery. Participants were asked if the delivery of the CBPET had altered their HWWS behaviors in their own homes. Twenty participants (91%) stated that “yes”, it had, with one participant not stating either way.

HWWS was just one stream of information embedded in the educational tool. Other key messages were the introduction of where germs came from and lived, how they travelled from host to recipient, how they gained access to the body and caused infection and when to wash your hands. Participants were asked if they had gained increased knowledge from the tool, and if so, to explain in their own words what they had gained. Twenty participants (91%) answered that they had gained more knowledge from the tool.

#### 3.4.1. Literacy

Participants explained they learned more from pictures than they did from words alone. Some participants were vocal in explaining that this had been the first time in their lives they could understand health-related information. Further analysis of their explanations revealed three separate thematic streams of new knowledge gained from the cartoon-based format of the tool: literacy, hygiene and transmission of germs. Another element that was communicated and well received was increased hygiene awareness, particularly that HWWS kills germs.

#### 3.4.2. Transmission of Germs

Possibly the most complex message to communicate via the CBPET was the transmission route of pathogens from host to recipient. Comments made by some participants indicated that this message was communicated successfully, with several people linking blood-borne contamination with hygiene and HWWS. If the CBPET tool can be successful in communicating the route of blood-borne disease in this small study, it could be employed in the fight against HIV and other sexually transmitted diseases across the rest of sub-Saharan Africa. This tool may assist with increasing the basic perception of pathogen transmission.

#### 3.4.3. Participants’ Comments on the Tool

The final question on the post-intervention questionnaire asked if any participant had a comment to make regarding the visual tool, and if so, to explain in their own words. Three themes could be identified from the comments made by twelve participants. Five participants (42%) requested that the short facilitator notes on the rear of the tool be translated into Swahili so more literate members of the community could deliver the tool themselves. Four participants (33%) wanted the tool distributed across a wider group so that more people could take advantage of the message it communicated, and three participants (25%) wanted to see more pictures in the tool.

## 4. Discussion

Poor adult literacy is a significant co-morbidity indicator [13,22]. Though there have been active global efforts to improve literacy levels [2], the impact remains significant in populations suffering from illiteracy [7,22]. Moreover, as poor literacy levels continue, communities with low literacy in low-resource settings continue to face health disparities, jeopardizing their health [7,14]. Hence, it would be more financially and logistically feasible to adapt the current methods by which healthcare messages are being delivered to ensure all communities are able to receive health information and reduce the health disparities among communities [7,9].

Developing healthcare education into a format that can be understood by adopting a universal language based on pictures or cartoons is the most practical way forward to ensure health equality among communities [9]. If key stakeholders can agree on a universal message system, health agencies can begin to educate the adult population that were previously unable to take advantage of global healthcare messages to promote health and well-being.

The CBPET did improve participants’ understanding of how germs are transmitted, and this increase in knowledge may start to produce a cultural change in regard to community practice and hygiene in the home. This may also lead to a HWWS culture that could prevent illness, disease and death [7,32,43,44,45,48]. Many developing communities with low access to health and community education continue to fall through public health educational gaps. Hence, the importance of providing a CBPET tool for communities suffering from low-literacy and low-resource is further highlighted to bridge the health literacy gap, equipping individuals with health knowledge that can lead to behavior change [7,14]. The results of this study indicate a suitably designed visual tool will provide the motivation and knowledge to improve HWWS. By educating more of the population on basic hygiene standards and promoting the washing of hands with soap and water, many lives could be saved from fecal–oral transmitted diseases.

### 4.1. Strengths and Limitations

One of the strengths of this study is tailoring the study methodology to ensure it is accessible to the community, such as utilizing an open-ended approach in collecting data. For example, though researchers prepared pre- and post-surveys, most surveys were conducted verbally, and participants answered based on their knowledge and understanding. This approach allowed participants to have a broad range of responses, tailoring it to the community context and literacy level. Besides that, this study was able to achieve translational impact, where the CBPET was utilized in the community in Tanzania and continues to be used in other settings too.

This study has several limitations. Firstly, due to the nature of the population with low literacy who had no formal education or limited education, the research team did not choose to include many questions besides their understanding of the CBPET to avoid burdening the participants. In addition, to suit the population’s needs, pre- and post-surveys were administered in an interview style. Hence, most results reported were descriptive, and conclusions were drawn from qualitative responses. Secondly, the sample size of this study is relatively small (N = 21) and mostly made up of men (96%). Though the study found that participants had an increase in health awareness and knowledge through CBPET, this result may not be generalizable to the greater population in Tanzania or all communities with low literacy. Thirdly, this study only focused on evaluating participants’ health knowledge after being provided with the CBPET. This study did not explore the long-term impacts of the CBPET due to funding, time and distance constraints.

### 4.2. Future Research Direction

Several research directions are suggested to further strengthen the literature gap of CBPET in communities with low literacy. Firstly, future research may evaluate the retention of health knowledge and the sustainability of behavioral changes using a pre-and-post study design with standardized quantitative measures to quantify the effectiveness of CBPET. This research direction would support quantifying the significance of CBPET in behavior changes such as HWWS.

Secondly, future research may focus on using certain cartoon elements that enhance knowledge retention or behavioral change. For instance, some research noted that the color of pictorial information [19], interactive elements [53] and tailored information [54] might impact knowledge retention. However, a recent systematic review did not observe any significant patterns in pictorial health interventions that exhibited an effect on knowledge retention and behavioral change [14], suggesting that these factors were not relevant in their effectiveness. Hence, future research is needed to explore the mechanism of the CBPET that promotes health awareness in communities with low literacy and poor resources.

## 5. Conclusions

Poor adult literacy is not just a characteristic of developing communities; it is a real threat in many developed nations globally as well. This study sought to determine if a new format to present vital healthcare information could bridge the literacy gap on a global scale. Disease and the spread of infection can be limited and controlled by employing basic hygiene standards such as handwashing with soap and water. The visual educational tool developed and evaluated for this study successfully educated a cohort with low textual literacy from a resource-poor setting to understand the basic chain of infection and the importance of washing hands with soap and water, as well as to show their intention for behavior-change. Further, the tool evaluated in this study showed potential for being a resource that community members could use to pass on accurate and consistent health messages without the need of health workers.

## Figures and Tables

**Table 1:** Religion, education level and occupation of the study group participants.

Characteristic	Sub-Category	N
Religion	Muslim	18
	Catholic	2
	Christian	1
Education level	Primary	17
	Secondary	3
	None	1
Occupation	Farmer	17
	Teacher	2
	Worker	1
	Driver	1

**Table 2:** Summary of participants’ hygiene knowledge.

Items	Participants’ Knowledge After Receiving CBPET
Knowledge of a germ	The number of participants identifying a fly as a germ halved from six (27%) to three (14%).
Knowledge of where germs live	The number of participants unable to explain where germs live reduced from five (15%) to two (6%).
Knowledge of germ transmission	The number of participants who understood how germs travel increased from 17 (77%) to 19 (86%).
Knowledge of germ infection	All participants (100%) reported understanding germ infection routes and provided a broader range of answers after receiving CBPET.
Previous education experience in handwashing	The number of participants who were confident they had been taught how to wash their hands increased from 13 (59%) to 18 (86%).
Understanding of the importance of handwashing with soap	All participants (100%) stated that handwashing was important after receiving CBPET.
Frequency of handwashing	The number of participants who intent to increase their handwashing frequency to seven+ times a day increased from three (14%) to five (27%).

## Data Availability

Data supporting reported results can be found with the first author, JB.
